# Helpers or halos: examining the evaluative mechanisms underlying selective prosociality

**DOI:** 10.1098/rsos.221188

**Published:** 2023-04-05

**Authors:** Kristen A. Dunfield, Laina Isler, Xiao Min Chang, Brandon Terrizzi, Jonathan Beier

**Affiliations:** ^1^ Department of Psychology, Concordia University, 7141 Sherbrooke Ouest, PY-146, Montreal, Quebec, Canada, H4B 1R6; ^2^ Department of Psychology, University of Toronto, Toronto, Canada; ^3^ Division of General and Community Pediatrics, Cincinnati Children's Hospital Medical Center, Cincinnati, OH, USA; ^4^ Independent Scholar, Washington, DC, USA

**Keywords:** altruism, reciprocity, partner choice, prosocial behaviour, social cognition

## Abstract

This research examines the proximate evaluative mechanisms underlying prosocial partner choice-based reciprocity. Across four studies we presented 855 university undergraduates (online for course credit) and 76 4- to 6-year-olds (offline at a university laboratory) with vignettes describing prosocial, social and non-social characters, and asked participants about their person preferences in prosocial, social and general contexts. Adults demonstrated sophisticated appraisals, coordinating between relevant trait and contextual cues to make selections. Adults were particularly attentive to prosocial cues in costly conditions, suggesting that they were using dispositional attributions to make their selections. By contrast, children were largely unable to integrate trait and contextual cues in determining their partner preferences, instead displaying valenced preferences for non-social cues, suggesting the use of affective tagging. Together, these studies demonstrate that the mechanisms underlying prosocial, partner choice-based reciprocity are not early emerging and stable but show considerable development over the lifespan.

## Highlights

— Proximate developmental mechanisms supporting partner choice are poorly understood
— Adults’ person preferences suggest they use dispositional evaluations
— Children's person preferences suggest they engage in affective tagging
— Mechanisms underlying mature partner choice develop over the lifespan

## 1. Introduction

Humans regularly act both *with* and *on behalf of* others. These other-oriented tendencies are thought to be foundational to our success as a species [1]. Despite its pervasiveness, however, a community's prosociality is vulnerable to individuals who seek to take advantage of others' good will. Prosocial actors must, therefore, meet two critical challenges: creating benefits for others and distributing those benefits sustainably [2]; consequently, it is believed that these two skills should emerge in tandem ([3], as cited in [2]).

Starting in the second year of life, children recognize and act on opportunities to benefit others in a diversity of ways that include helping, informing, sharing, comforting and cooperating (see [4,5] for reviews). Yet, the sustainable distribution of these benefits does not reliably emerge until age 4 [2,6]. In a series of four studies, we have examined how children (4- to 6-year-olds) and adults (university undergraduates) select between individuals who display characteristics ranging from prior prosocial tendencies (e.g. helpfulness) to positive but non-social traits (e.g. attractiveness), in domains ranging from prosocial investment (e.g. who would you help?) to random outcomes (e.g. who will win the lottery?). Together, these studies shed light on the development and nature of the proximate, evaluative mechanisms supporting human prosociality.

### 1.1. Distributing benefits

Acting on behalf of others can be costly. Effort, time and resources invested into another's well-being may bring no immediate rewards. When prosocial networks include too many *free riders*—individuals who take more than they give—they cannot be sustained [7]. How, then, do individuals maintain prosocial networks in a system so vulnerable to those who seek to take advantage? Trivers' theory of reciprocity [8] is a prominent solution whereby the immediate costs of prosociality are repaid in future interactions where roles are reversed. Under such conditions, individuals mutually benefit from engaging prosocially.

Current theories of reciprocity provide compelling ultimate explanations for costly prosociality. Yet, knowing how a behaviour can confer an adaptive benefit is not sufficient for a full understanding of that behaviour (e.g. [9]). Much less is known about the proximate mechanisms that underlie reciprocity or, importantly, how these mechanisms develop. By examining what information people draw on when choosing prosocial partners, the boundaries around these preferences, and the ways in which these preferences change over development, we gain important insight into humans’ extraordinary capacities for maintaining prosociality between unrelated individuals.

One way to maintain reciprocity is through partner choice [10]. Under partner choice models, individuals freely choose with whom to engage. Instead of focusing on the prevention of cheating, these models emphasize choosing and being chosen as a prosocial partner (e.g. [11–13]). Through partner choice, cooperation is rewarded by opportunities for interaction and non-cooperation is punished through exclusion. Partner choice-based reciprocity is observed relatively early in human development [14] and is prevalent in non-human animals (e.g. cleaner fish, [15]; see [2] for an exhaustive review).

### 1.2. Selective prosociality

There is a substantial literature demonstrating that, starting in infancy, people prefer prosocial characters over antisocial characters ([16–18]; for meta-analysis, see [19]) and preferentially invest their prosocial energies into those who have demonstrated prosocial intentions in the past (e.g. Adults: [20–22]; Children: [23–28]). Across a variety of assessments—from early in development through adulthood—people engage in selective prosociality in a manner consistent with critical features of partner-choice models: when there are two or more partners available, individuals prefer to engage prosocially with the partner demonstrating prosocial traits.

Starting in the first year of life, infants use valence, as opposed to perceptual characteristics to evaluate social interactions [29], prefer characters with a history of helping as opposed to hindering ([16], but see [30,31]) and expect others to hold similar preferences [32]. These expectations are not limited to helping and hindering interactions but generalize to fair and unfair givers as well [17,18]. When combined, a meta-analysis found that two thirds of infants (aged 4–32 months) preferred prosocial characters over antisocial characters [19]. Beyond these preferences, toddlers appear to generalize their observations to others expecting that individuals who engage in moral violations in one domain were less likely to follow moral principles in another [33]. Together, this research demonstrates that even very young infants are astute in their evaluations of others' behaviour and use these evaluations to guide their own behaviours and also make predictions about how others will interact.

As children age, these evaluations and preferences play out in their prosocial choices. For example, 21-month-olds preferentially help individuals who previously showed positive intentions to help them over those who had no intention to help, or accidentally provided help [23]. Shortly thereafter, children reciprocate prosocial intentions across diverse prosocial behaviours by helping fair [25], and informative [24] agents. Children have also been observed to invest in prosocial partners based on their behaviour towards others. Children preferentially share resources with those who have previously shared with others [26] and selectively help those who have previously helped others [27,28]. Through such selective prosocial behaviour, young children demonstrate their preference to help those who are prosocial and avoid interacting with those who are not, regardless of whether the past behaviour was directed towards themselves or others.

Yet, past prosociality is not the only factor associated with individuals’ prosocial motivations (see [6]). For example, adults are more prosocial towards people with whom they are familiar [34,35], or who share their group identity (e.g. [36,37]). Moreover, *non-prosocial* but reciprocal interactions (e.g. rolling a ball back and forth) can elicit prosocial behaviour in children [38]. Relatedly, socially relevant, but not prosocial cues such as imitation [39] and ostracism [40] have also been found to increase prosocial behaviour. Two-year-olds prefer to give an object to an individual who speaks their language over an individual who does not [41]. Four-year-olds share with their friends more than with peers or strangers [42,43], sometimes going so far as to prefer to share with friends over more needy others [44]. Five-year-olds prefer to give resources to those who share their gender, arbitrarily assigned group membership [45], and race [46]. Indeed, when these broader preferences are taken into consideration, it is not immediately clear that either adults or children are specifically preferring *prosocial* others leaving open important questions about the mechanisms, nature and development of the person preferences that support prosocial reciprocity.

### 1.3. Mechanisms underlying prosocial preferences

There is considerable debate regarding the mechanisms that underlie these prosocial preferences with explanations ranging from simple associative mechanisms [47] through to innate moral predispositions (e.g. [48]). Kuhlmeier *et al.* [6] have proposed three potential routes to prosocial partner choice, *valence matching*, *affective predictions* and *dispositional attributions,* each relying on a different set of proximate psychological mechanisms. The feature that distinguishes the first two routes (*valence matching*, *affective predictions*) from the third (*dispositional attributions*) is the type of evaluation that one individual forms about another. Under the first two ‘*affective tagging*’ routes, an evaluator attributes the valence of an observed behaviour to the individual producing the behaviour. By contrast, under the third route (dispositional attributions) costly prosociality is stabilized when individuals attribute *dispositions to* others based on observed behaviours.

Affective tagging could lead to reciprocity if individuals match the valence of interaction opportunities (helping = +) with the valence of the individual (helpers = +), acting positively toward a helper because they feel positively about them. Under the *valence matching* account, after an individual attributes valence to an actor they observed performing pro- or antisocial acts, they simply direct a similarly valenced action back (i.e. helping (+) a helper (+) or avoiding (−) an unfair distributor (−)). Previous research suggests infants have a precocious ability to detect such matches (e.g. [29]). Strict valence matching relies on the evaluation of immediate behaviours and does not require predictions about future actions.

*Affective predictions* work similarly to valence matching, except they involve predictions and/or expectations about the valence of an individual's future behaviour. That is, when an individual observes another engaging in pro- or antisocial behaviour, they not only attribute valence to that individual, but also predict that the individual will act in a consistent manner in the future (i.e. a fair distributor (+) will continue to act positively whereas a hinderer (−) will act negatively, e.g. [33]). Under this account, selective prosociality is supported by the expectation that positive individuals will continue to act positively in future interactions.

Although valence matching and affective predictions are proposed to be distinct mechanisms, they both rely on affectively tagging the observed individual. The present research cannot differentiate between these two specific mechanisms, thus they will be combined and referenced as ‘affective tagging’ accounts. Broadly, these affective tagging accounts have been used to explain why people prefer lucky individuals [49], how people identify good informants [50,51] and how non-human animals navigate the challenges of partner choice [52].

By contrast to affective tagging, dispositional attributions could lead to reciprocity by allowing an individual to make relatively specific predictions about what observed behaviours mean for likely future behaviours. Under dispositional attributions, observing an individual act prosocially leads an evaluator to make an attribution about the internal state or tendency (i.e. disposition) that caused the individual to act in a particular way [53]. For example, when observing an individual engage in helping behaviour, one might posit that they are not only a positive person, but that they are, more specifically, a nice or helpful individual leading to the prediction that they will act consistently in future. Whereas affective tagging leads to diffuse, valenced evaluations (e.g. the individual will continue to act positively), dispositional attributions include more specific predictions about the individual's likely future behaviour (e.g. they will continue to act prosocially).

There are few existing studies that can clarify whether individuals are using affective tagging or dispositional attributions when selecting prosocial partners, and how these mechanisms change over development. However, the different routes support different predictions about the conditions under which a person might prefer a specific partner. Because affective tagging mechanisms rely on evaluations that generate valence-based preferences, they may operate in contexts that do not specifically include prosociality. Consequently, individuals will not just preferentially help helpers; selective helping may also be triggered by other socially positive but non-diagnostic characteristics such as politeness, and even by non-social, non-diagnostic characteristics such as physical attractiveness (for a similar argument in the domain of selective social learning see [50,54,55]). Moreover, affective tagging mechanisms should not lead people to be uniquely selective in their prosocial interactions; their partner preferences should remain consistent across a diversity of positive, but non-prosocial interactions such as socializing. By contrast, under dispositional attribution mechanisms person preferences should reflect coordinated appraisals of trait and contextual cues, with a preference for prosocial partners becoming most pronounced in costly interactions.

To better understand the mechanisms that underlie partner choice-based reciprocity, it is critical to examine the boundaries around their operation, including how positive, non-prosocial characteristics influence prosocial decisions *and* how prosocial characteristics influence other social preferences. The current study will extend past research and examine how prosocial, social and non-social *traits* influence preferences in prosocial, social and non-social *contexts* over development.

### 1.4. The current investigation

Across four studies we used brief vignettes followed by forced-choice test questions to assess the person preferences of adults (university undergraduates) and children (4- to 6-year-olds). We chose to test children aged between 4 and 6 years old because children robustly direct prosocial behaviour to prosocial others starting at age 4 [2,6] and by 6 children can reliably attribute traits to others and use these traits to predict future behaviour [56,57]. To address limits of previous research, and better understand the mechanisms that support prosocial partner choice-based reciprocity, our vignettes described individuals who were prosocial (e.g. helpful), socially positive but not prosocial (e.g. polite) and positive but not social (e.g. attractive). Further, we asked our participants to make choices that were prosocial (e.g. who would you like to help?), social (e.g. who do you want to play with?) and non-social (e.g. who will win the lottery?). We asked adults (Experiment 1a) and children (Experiment 1b) who they preferred to interact with when selecting between individuals who demonstrated the positive and negative version of the same trait. If individuals are using affective tagging, we would expect them to consistently prefer the positive character, without attending to trait or context. If, however, individuals are using dispositional attributions, we should observe a preference for the positive prosocial individual, and heightened attentiveness to this relevant marker in the prosocial context. Experiments 2a (Adults) and 2b (Children) pushed the boundaries of these evaluations and asked participants to select between two characters who were both positively valenced, but varied on the type of positive trait displayed (e.g. one individual was helpful, whereas the other individual was attractive) across the three contexts. If individuals are using affective tagging, there should be no systematic preference regardless of trait or context because both individuals are positively valenced. By contrast, if individuals are using dispositional attributions, we should observe a preference for the prosocial character, particularly in the prosocial context. Further, we would expect an interaction whereby participants are especially likely to attend to prosocial cues when the context is costly, *and* the alternative character possesses irrelevant (non-social) traits. Finally, we predict that adults can and will use dispositional attributions, whereas children, who are still developing the ability to use past behaviour to make stable trait attributions [58], will be more likely to use affective tagging. The results of these studies will help to illuminate the proximate cognitive mechanisms that underlie partner choice-based reciprocity.

## 2. Method: Study 1a

### 2.1. Participants

Study 1a was conducted at two locations, Concordia University (QC, Canada), and the University of Maryland, College Park (MD, USA), using the same materials and procedure. Approval for recruitment strategies and experimental procedures were obtained from both research ethics boards. All participants were undergraduates, aged between 18 and 25 years. Sixty-seven participants (57 females, 1 not listed) were recruited from Concordia University and 363 (238 females) from the University of Maryland, College Park. The participants received course credits for participation. Five hundred and eighteen participants accessed the study: 21 were excluded for not completing, 37 were excluded for taking longer than 72 min (3 s.d. above the mean completion time), and 30 were excluded for completing the study in less than 10 min.

### 2.2. Materials

The experiment consisted of 72 items, 24 describing Prosocial traits (12 Helpful, 12 Generous), 24 Social traits (12 Prestigious, 12 Considerate) and 24 Non-social traits (12 Attractive, 12 Intelligent). For each item, participants first learned about two characters varying on the same trait. Then, participants learned about a context in which they could choose between the two characters. Half of the trials described female characters and half described male characters. The order of valence presentation was counterbalanced. All materials are available in OSF (https://osf.io/e4zyf/).

Trials were generated by drawing from a set of four stems depicting a scenario in which each trait could be expressed. We created positive and negative versions of each stem (e.g. John is helpful and glad to assist others; Tom is unhelpful and never assists others). To generate the 12 test items per trait, we paired each positive stem with the negative versions of the other three stems for that same trait.

Each trial concluded with two test questions presented in a fixed order. First, participants chose between the two characters, in one of three contexts: Prosocial (i.e. Suppose both John and Stewart are each working on different projects for class. You know a great time-saving technique, but only one person can use it. Who would you tell?), Social (i.e. Suppose both Kelly and Mila are travelling separately on a long train ride. There is an open seat next to each one. Who would you prefer to sit beside?) or General (i.e. Suppose both Jessica and Elsa have entered a lottery that you are running. You are blindfolded and reach into the bowl to pull out a name. Whose name are you more likely to draw?). Second, participants rated their confidence in that choice on a 5-point Likert-scale from ‘Not confident at all’ to ‘Very confident’. Because we are using a force choice method, we were concerned that participants' selections might overstate their actual preferences. By asking participants to rate their confidence in their judgements, we hoped to gain a slightly more nuanced picture of their preferences. The three varieties of forced-choice questions were distributed across items such that each one was paired with each positive stem and each negative stem the same number of times. Table 1*a* in the electronic supplementary material provides examples of trait stimuli and context test questions.

**Table 1.** Main effects model estimates for adults' positive person preference at each level of Trait (top panel) and Context (bottom panel). Note: for pairwise comparisons view electronic supplementary material, table S3a.

	beta	s.e.	*Z*	*p*-value	OR
Trait					
Non-social	0.72	0.16	4.49	<0.001	2.06
Social	1.82	0.16	11.21	<0.001	6.18
Prosocial	2.80	0.17	16.16	<0.001	16.46
Context					
General	1.09	0.16	6.83	<0.001	2.97
Social	2.54	0.16	15.62	<0.001	12.68
Prosocial	1.71	0.16	10.58	<0.001	5.53

### 2.3. Procedure

Participants completed this online study independently using the SONA platform and provided informed consent before starting. Upon completion, participants were debriefed about the nature and purpose of this study. The full battery of items took 15–20 min to complete.

### 2.4. Analytic strategy

Choices were coded such that choosing the positive character was a ‘hit’ (1) and choosing the negative character was a ‘miss’ (0). Person preferences were analysed using generalized linear mixed models (GLMMs) implemented in the lme4 package (v.1.1.29) in R (v.4.2.1). Model estimates were generated using maximum likelihood via Laplace approximation and a logit link function. Follow up tests (see electronic supplemental material) were conducted using the emmeans (v.1.7.5) and multcomp (v.1.4.19) packages using Tukey's correction for multiple comparisons. Participants' confidence ratings were assessed using a linear mixed model including these same fixed and interaction effects.

We generated two models for the primary analyses. Model 1 included random intercepts for Participant ID and Item Number to capture variance attributed to the randomness of subject and item sampling. We then used a data-driven approach to generate Model 2. We began with a main effects only model of Trait and Context, and then assessed the comparative model fit after adding an interaction between the two predictors. Random slopes of Trait were included in both levels of Model 2, along with the previously described intercepts. Notably, the interaction model explained significantly more variance in participants’ responses than a main effects only model ($\chi_{4}^{2}=46.40$, *p* < 0.001).

### 2.5. Results: Study 1a

#### 2.5.1. Person preference

**Figure 1.**
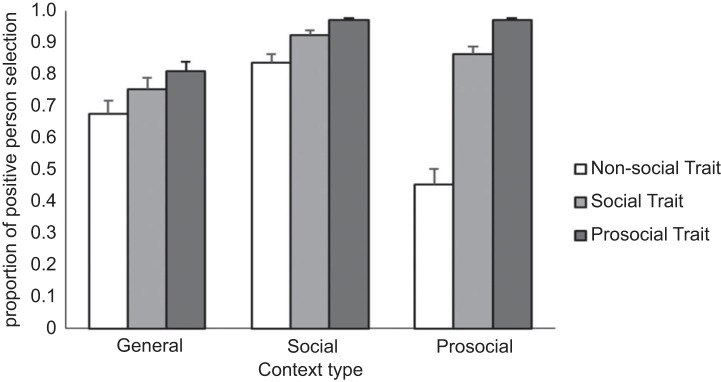
Proportion of adult participants selecting the positive character at each level of Context and Trait. Standard error bars indicate 95% CIs. Note: for simple main effects and follow-up contrasts, view electronic supplementary material, tables S3b and S3c, respectively.

Model 1 (random intercepts only) revealed that across all test items, the odds of choosing the positive character were approximately five times greater than those of choosing the negative character (*b* = 1.64, s.e. = 0.15, *z* = 11.22, *p* < 0.001; OR = 5.16). The main effects model demonstrated that participants selected the positive character over the negative character across every description of Trait and Context (table 1), with the greatest preference observed for the Prosocial character, and the weakest for the Non-social character. However, this effect was qualified by an interaction (figure 1).

Follow up contrasts reveal that in the Prosocial and Social contexts, the effects of all three traits (Prosocial, Social, Non-social) differed, whereas in the General context, only the Prosocial and Non-social trait effects differed. Additionally, the observed preference for the positive Prosocial individual, relative to the positive Non-social individual, was weakest in the General context, with only a two-fold increase in odds (OR = 2.03, *p* = 0.025), and strongest in the Prosocial context (OR = 40.41, *p* < 0.001), wherein the odds were 40 times greater. Further, in the Prosocial context, participants did not show any preference for the positive Non-social character (OR = .83, *p* = 0.335).

#### 2.5.2. Confidence ratings

**Figure 2.**
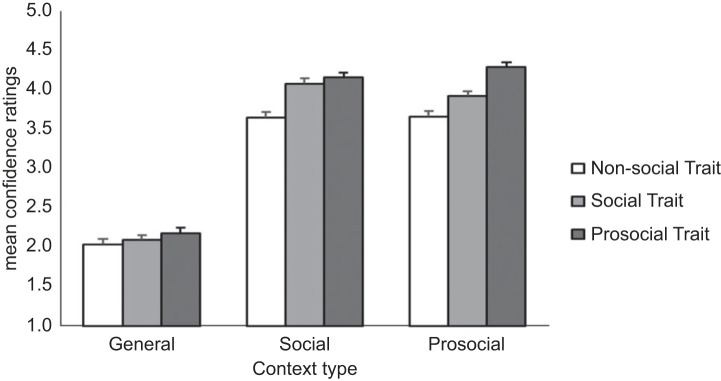
Adults' mean confidence ratings by Context and Trait. Standard error bars indicate 95% CIs. Note: for simple main effects and follow-up contrasts, view electronic supplementary material, tables S4b and S4c, respectively.

The main effects model predicting confidence in character selections demonstrated that, excluding the General context, participants were confident in their person preferences (i.e. above mid-point; table 2). Additionally, participants were most confident when assessing Prosocial characters, and least confident when assessing Non-social characters. This effect was qualified by an interaction ($\chi_{4}^{2}=22.64$, *p* < 0.001; figure 2). Specifically, trait did not affect confidence in person preferences in the General context, while confidence in character selection was equivalently strong when considering Social and Prosocial characters within the Social context.

**Table 2.** Main effect model estimates for adults’ confidence in their person preferences at each level of Alternative Trait (top panel) and Context (bottom panel). Note: for pairwise comparisons view electronic supplementary material, table S4a.

	mean	s.e.	*Z*	*p*-value
Trait				
Non-social	3.12	0.05	63.88	<0.001
Social	3.36	0.05	68.95	<0.001
Prosocial	3.55	0.05	72.67	<0.001
Context				
General	2.11	0.05	43.18	<0.001
Social	3.96	0.05	81.22	<0.001
Prosocial	3.96	0.05	81.09	<0.001

### 2.6. Discussion: Study 1a

Study 1a examined factors that affect adults' person preferences across different traits and contexts. Overall, participants were sensitive to the valence of past behaviour, preferring to interact with positive over negative individuals. However, these preferences were qualified in several ways. First, adults were influenced by the nature of the trait, most strongly preferring individuals with a history of positive prosocial behaviour. Second, adults were influenced by the context of their choices, demonstrating the highest overall preference for the positive characters in the social context. Finally, we found that participants were sensitive to the *interaction* between trait and context. Specifically, adults showed the greatest range in person preferences in the prosocial context, wherein they demonstrated a strong preference for the positive prosocial individual and no preference for the positive non-social individual. Further, participants' confidence judgements were consistent with these preferences. Participants were not confident in their selections in the general context, and least confident in their judgements of non-social traits. Together, this suggests that adults are using something like dispositional attributions when selecting between potential partners, as valence-based evaluations would not result in the observed coordination between displayed trait and decision context. Study 1b uses a similar design to examine the development of these preferences in children.

## 3. Study 1b

### 3.1. Participants

Forty-eight children participated in this study: twenty-four 4-year-olds (12 females, *M*_age_ = 48.37 months, range = 42–54 months), and twenty-four 6-year-olds (*N* = 24, 13 females, *M*_age_ = 72.08, range = 66–77 months). Participants were recruited from Montreal, QC, Canada. Participants’ cultural and language backgrounds were diverse, but all children understood and spoke English as their first or second language. Five additional participants were tested but excluded from data analysis due to incorrectly answering half of the memory checks (2), experimenter error (1) or participant-initiated termination before study completion (2).

### 3.2. Materials

The stimuli consisted of 18 items, six describing Prosocial traits (Helpful), six Social traits (Polite) and six Non-social traits (Attractive). Children viewed pairs of colourful drawings, depicting children who differed on the valence of a single trait. As with Study 1a, participants were asked to choose a character in three contexts: Prosocial, Social or General. Characters had unique appearances (i.e. hair colour, clothing colour) and names, and matched the gender of the participant (see https://osf.io/e4zyf/ for full stimuli). Electronic supplementary material, table S1*b* provides examples of the trait stimuli and context test questions.

### 3.3. Procedure

Following warm up and consent, a female experimenter took the child into the testing room. Parents who accompanied their children into the testing room were seated out of view of the child and asked to keep silent. No participants were excluded due to parental interference.

The experimenter and the participant sat at a desk facing each other. On each trial, the experimenter presented the participant with a pair of character illustrations then introduced and described the characters (e.g. ‘This is Lily, Lily is very helpful. Look, here she is picking up this toy. This is Jasmine, Jasmine is not very helpful. Look, here she is ignoring this toy.’). The order of presentation was counterbalanced across participants. Participants were asked four questions that reflected the three different Contexts: Prosocial (Who would you like to help?), Social (Who would you like to play with?) and General (Imagine you have a cookie and a piece of broccoli. Who would you like to give the cookie to? Lily or Jasmine? Who would you like to give the broccoli to? Lily or Jasmine?). The illustrations of both characters were visible to the child for the full duration of a trial. The order of the test questions was pseudo-randomized across trials. Lastly, participants received a memory check (i.e. ‘Who was helpful (polite, pretty/handsome)? Lily or Jasmine? Who was not helpful (polite, pretty/handsome)?’ Lily or Jasmine?). Participants who incorrectly answered more than half of the memory check items were excluded from analysis and replaced. After completing the test trials, participants were asked ‘What do you prefer? Cookie or broccoli?’. This question was designed to identify the participant's food preference, which would affect their responses to the two food assignment questions. The General (food assignment) context was intended to ensure that participants were not perseverating on a single response or avoiding the negatively valenced character. Moreover, we were concerned about the youngest participants' ability to understand ‘random’. Although we recorded both choices, they were mutually exclusive, and we only analysed the preferred option. The entire experiment took 10–12 min to complete. The child received a certificate and a small toy for their time.

### 3.4. Analytic strategy

Following Study 1a, choosing the positive character was coded as a ‘hit’ (1) and choosing the negative character was coded as a ‘miss’ (0). Again, we generated two models. Model 1 included only the random intercepts of Participant ID and Item Number. We then used a data-driven approach to generate Model 2. Initial results demonstrated no improvement in fit when incorporating an interaction between Trait and Context against a main effects only model ($\chi_{4}^{2}=1.07$, *p* = 0.900). We next considered the significance of each main effect in turn. The inclusion of Trait as a fixed effect demonstrated a superior fit over the random intercepts model ($\chi_{1}^{2}=13.57$, *p* < 0.001). Moreover, adding the fixed effect of Context accounted for further variance in the model ($\chi_{2}^{2}=17.12$, *p* < 0.001). Low variability associated with item intercepts, as well as slope of trait, resulted in a singular fit, thus the model was simplified to account for only the random intercepts of Participant.

### 3.5. Results 1b

#### 3.5.1. Memory check

Overall, participants correctly identified both the positive and negative individual on 96.60% of the trials (4-year-olds *M* = 94.58%, s.d. = 8.7; 7-year-olds *M* = 98.61%, s.d. = 4.7).

#### 3.5.2. Person preference

Model 1 (random intercepts only) revealed that at baseline the odds of choosing the positive character were almost six times greater than those of choosing the negative character (*b* = 1.76, s.e. = 0.25, *z* = 7.15, *p* < 0.001, OR = 5.83).

Review of the main effects demonstrated that the positive character was preferred across all categories of Trait and Context (table 3). However, the preference for the positive character was greatest when considering the Non-social trait (i.e. Attractiveness). Specifically, the odds of selecting the positive Non-social character were three times those of the Socially positive character (OR = 3.34, *p* < 0.001), and almost twice those of the Prosocial character (OR = 1.84, *p* = 0.043). By contrast, participants were least likely to select the positive character within the General context. Specifically, the odds of selecting the positive character were more than two times greater in both Prosocial (OR = 2.18, *p* = 0.002) and Social (OR = 2.45, *p* < 0.001) contexts, as compared to the General context.

**Table 3.** Model estimates for children's positive person preference at each level of Trait (top panel) and Context (bottom panel). Note: for pairwise comparisons view electronic supplementary material, table S5a. We investigated the potential role of fatigue and did not find evidence that first trial effects differed substantively from the observed pattern, see table electronic supplementary material, table S5b.

	beta	s.e.	*Z*	*p*-value	OR
Trait					
Non-social	2.42	0.28	8.50	<0.001	11.25
Social	1.21	0.25	4.88	<0.001	3.35
Prosocial	1.81	0.26	6.93	<0.001	6.11
Context					
General	1.25	0.25	5.04	<0.001	3.49
Social	2.15	0.27	7.86	<0.001	8.58
Prosocial	2.03	0.27	7.56	<0.001	7.61

#### 3.5.3. Age comparison

To assess developmental change, we considered age as a potential moderator. Age was added as a main effect to the previously described fixed effects model. Results demonstrated a significant effect of age, (*b* = 1.09, s.e. = 0.40, *z* = 2.70, *p* = 0.007, OR = 2.98), whereby the odds of selecting the positive character were three times greater among 6-year-olds as compared to 4-year-olds. Subsequent models then individually added interaction terms of Age and Context, and of Age and Trait, in turn. Neither the interaction of Age and Context ($\chi_{2}^{2}=1.75$, *p* = 0.417), nor Age and Trait ($\chi_{2}^{2}=4.34$, *p* = 0.114) reached significance over a main effects only model. However, because the Age by Trait interaction was (i) hypothesized *a priori*, (ii) trended towards significance and (iii) likely lacked statistical power due to a relatively small sample size (*N* = 48), we explored the resultant pattern.

**Figure 3.**
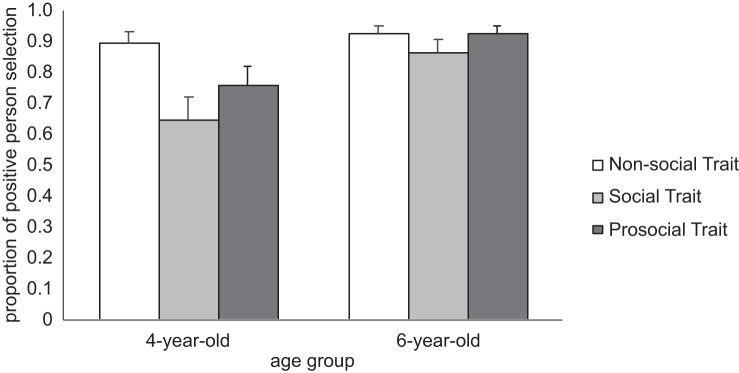
Proportion of younger and older children selecting the positive character at each level of Trait. Standard error bars indicate 95% CIs. Note: for simple main effects and follow-up contrasts, view electronic supplementary, material, tables S5b and S5c, respectively.

Follow up contrasts demonstrated that 6- and 4-year-olds do not differ in their preference for the positive character when considering the Non-social trait (OR = 1.47, *p* = 0.466). However, 6-year-olds were more likely than 4-year-olds to choose the positive character when considering the Social (OR = 3.54, *p* = 0.007) and Prosocial (OR = 4.11, *p* = 0.005) traits (figure 3). When assessed independently, 6-year-olds demonstrated equivalently strong preferences when considering positive Prosocial and Non-social characters (OR = 12.81, *p* < 0.001).

### 3.6. Discussion: Study 1b

Study 1b examined the factors that affect children's person preferences. Like adults, children were sensitive to the valence of past behaviour, preferring to interact with positive over negative individuals and the strength of this preference varied based on the trait being considered. Unlike adults, children appeared to have the strongest preference for individuals displaying positive non-social traits (i.e. attractiveness). Importantly, the relative preference for the positive non-social characters over the two socially positive characters lessened with age. That is, relative to 4-year-olds, 6-year-olds showed a stronger preference for the prosocial and socially positive characters, suggesting development towards the more mature person preferences. Further, like adults, children were attending to the context, showing a greater sensitivity to trait valence in the two social contexts (social and prosocial) than the general context. Finally, we found no interaction between trait type and context, suggesting that at this age, children do not integrate these considerations and are likely using a valence-based selection strategy.

## 4. Studies 2a and 2b

Studies 1a and 1b demonstrate that both children and adults prefer positive individuals across a variety of traits and in a diversity of contexts. The strength of this preference varied depending on the age of the participant and the specific trait under consideration. Adults were choosiest when considering prosocial individuals across all contexts. Moreover, consistent with dispositional attributions, this trait effect was most pronounced in the prosocial context. By contrast, children showed the strongest preference for the positive non-social individual, regardless of context, and this preference lessened with age.

On the one hand, it is consistent with theories of reciprocity that our adult participants demonstrated more complex character appraisal patterns in the prosocial context, ignoring non-social cues, while maintaining a high focus on prosocial tendencies. On the other hand, it is surprising and inconsistent with theories of precocious partner choice that children's strongest person preference was for the non-social individual, for whom the children had no information regarding behavioural tendencies.

Ceteris paribus, preferring positive characters, or avoiding negative characters, is not surprising. Indeed, in the absence of diagnostic information, affective tagging (preferring positive individuals for positive interactions) is a sensible strategy. Studies 2a and 2b will extend these findings and probe the specificity of the evaluative mechanisms by examining how individuals select between two partners who are both positively valenced but differ regarding the trait within which they display their positivity.

## 5. Method: Study 2a

### 5.1. Participants

Study 2a was also conducted at Concordia University and the University of Maryland, College Park, using the same materials and procedures as Study 1a. All the participants were undergraduates, aged between 18 and 25 years old. One hundred and three participants (92 females, 1 not listed) were recruited from Concordia University and 322 (226 females, 3 not listed) from University of Maryland, College Park. Participants were granted course credits for their participation. Four hundred and ninety-six participants accessed the study: 23 were excluded for not completing, 36 were excluded for taking longer than 72 min (over 3 s.d. above the mean completion time) and 12 were excluded for taking less than 10 min to complete the study.

### 5.2. Materials

The experiment consisted of 72 item pairings. In each pairing, a Prosocial (Helpful or Generous) character was compared to a positively valenced alternative. In half the pairings, this alternative was Social (18 Prestigious, 18 Considerate), and in the other half, this alternative was Non-social (18 Attractive, 18 Intelligent). For each pairing, participants learned about the two characters varying on the different positive traits. Then, participants learned about a context (Prosocial, Social, General) in which they could choose between the two characters.

As with Study 1a, trials were gender balanced. The order of Prosocial versus alternative characteristic being presented first was counterbalanced. Each vignette was followed by two test questions presented in a fixed order: a forced-choice character selection, and a confidence rating on a 5-point Likert-scale. The three types of test questions were evenly distributed across different characteristic pairs. Although we assessed within-trait pairings of the Prosocial characters, we were specifically interested in those items contrasting Prosocial and alternative (i.e. Social or Non-social) characteristics. See https://osf.io/e4zyf/ for complete stimuli. Table S2a in the electronic supplementary material provide examples of the trait contrast stimuli.

### 5.3. Procedure

Participants completed this study entirely online in the same manner as Study 1a.

### 5.4. Analytic strategy

Again, we generated two models. Model 1 was an ‘empty’ model containing only Participant ID and Item Number as random intercepts. Model 2 was generated using the stepwise approach previously described and compared main effects and interaction models of Alternative Trait and Context. Random slopes of Alternative Trait were also included in both examples of Model 2. The interaction model provided a better fit than a main effects only model ($\chi_{2}^{2}=11.49$ , *p* = 0.003).

### 5.5. Results: Study 2a

#### 5.5.1. Person preference

Model 1 (random intercepts only) indicated that across all test items, the odds of choosing the Prosocial character were approximately twice those of choosing the alternative (*b* = .82, s.e. = 0.08, *z* = 9.85, *p* < 0.001; OR = 2.27). Additionally, results from the main effects model revealed that participants selected the Prosocial character over *either* alternative character choice, and in every Context category (table 4). Notably, the preference for the Prosocial character was particularly strong when the alternative was described as having Non-social, rather than Social, traits (OR = 1.67, *p* < 0.001).

**Table 4.** Main effects model estimates for adults' prosocial person preference at each level of Alternate Trait (top panel) and Context (bottom panel). Note: for pairwise comparisons view electronic supplementary material, table S6a.

	beta	s.e.	*Z*	*p*-value	OR
Alternate Trait					
Non-social	1.10	0.08	13.60	<0.001	3.01
Social Trait	0.59	0.07	8.15	<0.001	1.81
Context					
General	0.71	0.09	8.07	<0.001	2.03
Social	0.38	0.09	4.29	<0.001	1.45
Prosocial	1.46	0.09	16.50	<0.001	4.31

**Figure 4.**
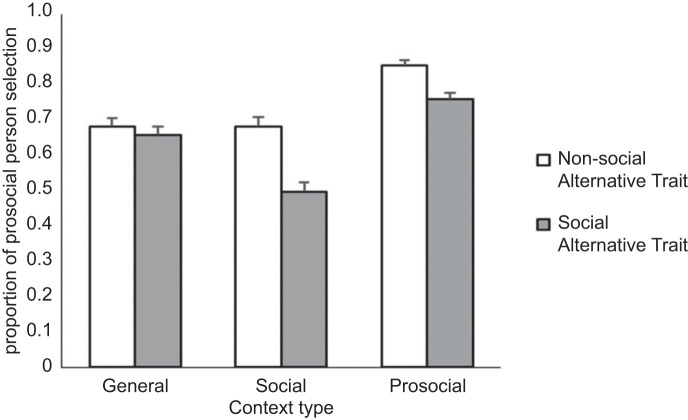
Proportion of adult participants selecting the prosocial character by Context and Alternative Character Trait. Error bars are 95% CIs. Note: for simple main effects and follow-up contrasts, view electronic supplementary material, tables S6b and S6c, respectively.

Follow up contrasts of the interaction model (figure 4) further clarified this trait effect. Specifically, this *relative increase* in preference for the Prosocial character over a Non-social (rather than Social) alternative, was not observed within the General context (OR = 1.11, *p* = 0.460), and was strongest within the Social context (OR = 2.20, *p* < 0.001). Unpacking this pattern, we found no effect of favouring the Prosocial character over the Social alternative within the Social context (OR = .98, *p* = 0.866). Moreover, the largest preference for the Prosocial trait was found when contrasting the Prosocial character to a Non-social alternative within the Prosocial context (OR = 5.94, *p* < 0.001).

#### 5.5.2. Confidence ratings

**Figure 5.**
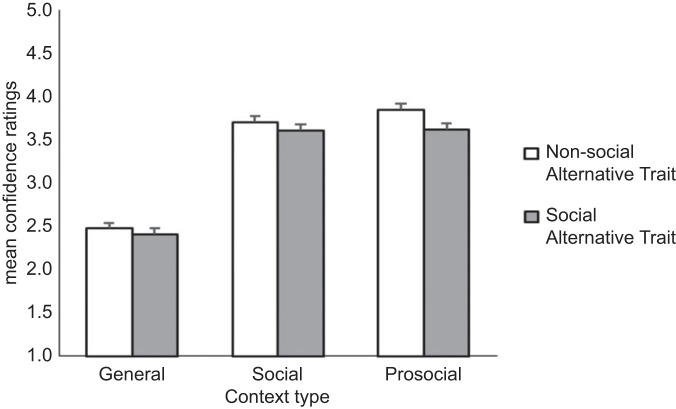
Adults’ mean confidence ratings for Context and each Alternative Character Trait. Error bars are 95% CIs. Note: for simple main effects and follow-up contrasts, view electronic supplementary material, tables S7b and S7c, respectively.

As with Study 1a, outside of the General context, participants were confident in their character selection. Analogous to Study 1a, where participants were most confident evaluating Prosocial characters, and least confident evaluating Non-social characters, participants were most confident when evaluating a Prosocial character paired with a Non-social alternative (table 5). This effect was qualified by an interaction ($\chi_{2}^{2}=18.71$, *p* < 0.001; figure 5) whereby the *increased* confidence observed when the Prosocial character was paired with a Non-social (rather than Social) alternative was greatest in the Prosocial condition .

**Table 5.** Main effects model estimates for adults’ confidence at each level of Alternate Trait (top panel) and Context (bottom panel). Note: for pairwise comparisons view electronic supplementary material, table S7a.

	mean	s.e.	*Z*	*p*-value
Alternate Trait				
Non-social	3.34	0.03	100.29	<0.001
Social	3.21	0.03	96.38	<0.001
Context				
General/Random	2.45	0.03	70.96	<0.001
Social	3.56	0.03	105.98	<0.001
Prosocial	3.73	0.03	108.28	<0.001

### 5.6. Discussion: Study 2a

Study 2a extends the results of Study 1a by examining adults' person preferences in the absence of valence cues. When selecting between two positively valenced individuals, participants demonstrated the ability to take specific characteristics into consideration, showing a strong overall preference for the prosocial character. Participants also showed sensitivity to context. Specifically, in the social context, both prosocial and social individuals were preferred equally. This is sensible because both traits signal the individual would be nice to socialize with. Further, the preference for the prosocial over either alternative was strongest in the prosocial context, most notably when contrasted against the non-social alternative. Indeed, in this more specific social context (i.e. who to help), the two social traits (i.e. prosocial and social) are *not* equally good at signalling future prosocial intent. Moreover, the latter effect mirrors results from Study 1a, in which the strongest contrast effect was found when comparing preferences between non-social and prosocial traits in the prosocial context. Finally, participants' confidence ratings suggest that these evaluations are being made thoughtfully taking into consideration the relevance of the information provided. Specifically, participants expressed that they lacked confidence in the general context and were most confident when considering prosocial traits paired with non-social alternatives.

Taken together, Studies 1a and 2a provide evidence that adults' reciprocity is supported by attending to both traits and contexts and flexibly selecting partners based on the joint consideration of these factors. Together, these studies suggest that adults are using something like dispositional attributions to identify good partners and stabilize costly prosociality. Study 2b extends this examination to children.

## 6. Method: Study 2b

### 6.1. Participants

Twenty-eight 6-year-old children (14 girls) participated in this study (*M*_age_ = 70.29 months, s.d. = 4.84, Range = 66–87 months). Participants were recruited from Montreal, QC, Canada. Participants’ cultural and language backgrounds were diverse; however, all children understood and spoke English as their first or second language. Eleven additional participants were excluded from analysis for experimenter error (1), failure to comprehend (2), inattention (1), withdrawing assent (1) and incorrectly answering half of the memory checks (6).

We collected data from 35 4-year-olds; however, we were unable to analyse data for 21 of these participants due to failure to complete the study (3), difficulties with language comprehension (3) and incorrectly answering more than half of the memory checks (15). Because almost half of the younger sample was excluded for failing to correctly identify the trait associated with each character, despite having the images visible, we made the decision not to analyse the data from this age group.

### 6.2. Materials

Children viewed four pairs of coloured drawings. In each pairing, a Prosocial (Helpful) child was compared to a positively valenced alternative. In two pairings, this alternative was Social (Polite), and in two, the alternative was Non-social (Attractive). As with Study 2a, participants were asked to select between the two individuals in three contexts: Prosocial, Social or General. All characters had unique appearances (i.e. hair colour, clothing colour) and names, and matched the gender of the participant. Although Social and Non-social pairings were also presented, we only analysed trials which included our Prosocial target. Electronic supplementary material, table S2b provides examples of the trait contrast stimuli.

### 6.3. Procedure

Study 2b used the same stimuli and procedure as Study 1b.

### 6.4. Analytic strategy

Choosing the Prosocial character resulted in a ‘hit’ (1) and choosing the alternative character resulted in a ‘miss’ (0). We generated two models: Model 1 included only the random intercepts of Participant ID and Item Number. Again, we used a data-driven approach to generate Model 2. Initial results demonstrated no improvement in fit when incorporating an interaction between Alternative Trait and Context against a main effects model ($\chi_{2}^{2}=0.18$, *p* = 0.914). We next considered the significance of each main effect in turn. The inclusion of Alternative Trait as a fixed effect did not result in a superior fit to an intercept only model ($\chi_{2}^{2}=4.11$, *p* = 0.128), suggesting that the specific nature of the alternative character (Social or Non-social) did not influence participants' preferences. However, the inclusion of Context predicted preference for the Prosocial over the Alternative, while controlling for the effect of Trait ($\chi_{2}^{2}=6.82$, *p* = 0.033). Each fixed effect model included random intercept and slope of Alternative Trait. However, the inclusion of random intercept of Context resulted in a singular fit due to low variance and was omitted from the analyses.

### 6.5. Results Study 2b

#### 6.5.1. Memory check

Overall, 6-year-olds were able to correctly remember which positive traits were associated with which character, consistently responding correctly to the memory check questions (*M* = 87.38%, s.d. = 14.12).

#### 6.5.2. Person preference

Model 1 (random intercepts only) indicated that participants were not significantly more likely to choose the Prosocial character over the alternative (*b* = 0.27, s.e. = 0.16, Z = 1.67, *p* = 0.094, OR = 1.31). Moreover, the nature of the Alternative Trait did not predict participant choice. Consistent with these findings, and despite a fractional (one-third) increase in odds, the participants were not significantly more likely to prefer the Prosocial character over either alternative. Finally, when considering the role of context, the only significant effect was found in the General context, such that participants were most likely to choose the Prosocial character over the alternative when assigning a preferred food (table 6).

**Table 6.** Main effects model estimates for children's prosocial person preference at each level of Alternate Trait (top panel) and Context (bottom panel). Note: for pairwise comparisons view electronic supplementary material, table S8a. We investigated the potential role of fatigue and did not find evidence that first trial effects differed substantively from the observed pattern, see electronic supplementary material, table S8b.

	beta	s.e.	*Z*	*p*-value	OR
Alternate Trait					
Non-social	0.28	0.24	1.16	0.244	1.32
Social Trait	0.30	0.24	1.26	0.209	1.36
Context					
General	0.66	0.25	2.64	0.008	1.93
Social	−0.16	0.24	0.65	0.519	0.86
Prosocial	0.36	0.24	1.48	0.140	1.43

### 6.6. Discussion Study 2b

The results of Study 2b complement the previous three studies and help to illuminate the mechanisms underlying partner choice-based reciprocity. Specifically, Studies 1a and 2a demonstrated that adults are highly sensitive to the specific content of the information they receive about others when selecting prosocial partners. Study 1b found that over the course of development, children become increasingly aware of the relevance of social information and move from a heavy bias towards superficial valence information (e.g. attractiveness) towards more meaningful social information (e.g. prosociality). Yet, in the current study, we found that in absence of contrasting valence information, 6-year-olds struggle to use relevant, available information to select partners for interaction. Moreover, it is striking that many 4-year-olds failed to correctly identify the traits associated with each character despite having these traits labelled *and* having the corresponding images available. The exclusion of the 4-year-olds in Study 2b, combined with the strong preference for the attractive individual in Study 1b, speaks to the difficulty children at this age appear to have ascribing consistency to others’ behaviour (e.g. [59]). When taken together, Studies 1b and 2b suggest that children are neither attending to, nor using trait information in the ways adults do, instead children appear to be engaging in selective prosociality based on a less thoughtful, affective tagging strategy.

## 7. General discussion

Human prosociality requires individuals to create benefits for others, and sustainably distribute those benefits [2]. Reciprocity—preferentially helping those who have helped in the past—is an ultimate explanation for how individuals can navigate the risks of prosociality while exploiting the benefits [8]. Yet, ultimate explanations fail to illuminate proximate considerations (i.e. mechanism and ontogeny). The current research explored the development of prosocial partner choice by examining the evaluations and preferences that support reciprocity across development. Specifically, we examined whether and when individuals use affective tagging versus dispositional attributions to choose their prosocial partners. Under affective tagging, individuals should generally prefer others with a history of positive behaviour regardless of the nature of the positive trait or context under consideration, under dispositional attributions, person preferences should be relatively specific to prosocial individuals in prosocial contexts.

### 7.1. Adults: dispositional attributions

Study 1a examined adults' valence-based preferences across three types of traits—prosocial, social and non-social—and three contexts—prosocial, social and general. Consistent with dispositional attributions, we found that adults show the strongest preference for positive prosocial individuals, especially when considering individuals within the prosocial context.

Study 2a further asked how individuals make these selections in the absence of valence information. In this more challenging evaluative task, adults showed coordinated appraisals of trait and contextual cues. Consistent with dispositional attributions, adults were particularly attentive to prosocial cues in the prosocial context, and the only condition in which adults did not display a preference for the prosocial character was when they were paired with a socially positive alternative in the social context. This pattern of results demonstrates the sensitivity of adult's preferences as both social and prosocial traits signal the likelihood of a positive social interaction. Despite the limited information we presented, adults are highly attuned to the relevance of trait information when determining how to invest their prosocial energies.

Taken together, these two studies provide strong support for the claim that adults maintain reciprocal prosocial interactions through the evaluation of, and preference for, individuals demonstrating diagnostically relevant information. The interaction between trait and context in the selection of partners strongly suggests that adults are using something like dispositional attributions when determining where to invest their prosocial energies, allowing them to exploit the benefits of prosociality while effectively mitigating the risks posed by free riders.

### 7.2. Children: affective tagging

A common interpretation of past research is that children's selective prosociality is astute and precocious (e.g. [24,60]), yet our results suggest that the mechanisms underlying children's prosocial person preferences are neither as sophisticated nor as specific as adults'. If children were using dispositional attributions to identify and select their prosocial partners, we would expect to see a preference for those displaying positive prosocial traits, especially when making decisions in a prosocial context. Although children showed a global preference for positive characters over negative characters (Study 1b), the traits they emphasized differed from those prioritized by adults, and those that would be expected if the children were making dispositional attributions (i.e. prosocial traits). That is, in Study 1b children showed the strongest preference for the positive *non-social* character. Moreover, unlike adults, these preferences did not interact with the context in which the selections were being made. Critically, these results cannot be attributed to misremembering/identifying the characters, as participants at both ages were highly accurate in remembering the valence of each character.

Study 2b further qualified these results. In the absence of valence information, 6-year-olds failed to show a reliable preference for the prosocial character, regardless of which alternative they were paired with. Furthermore, 6-year-olds showed little sensitivity to context. Surprisingly, it was *only* in the general context that the 6-year-olds showed a clear preference for the prosocial individual. Together these results suggest that the mechanisms underlying partner choice-based reciprocity mature over the course of development beginning with a preference for non-mentalistic, positive information and only later recruiting associated social cognitive abilities, such as trait attributions, to make more appropriate selections.

These results are consistent with past work on the development of behaviour-to-trait attributions. Specifically, although 4-year-olds can form evaluative judgements after a single exposure, they typically require linguistic scaffolding (e.g. explicit trait language) to make the appropriate behaviour to trait attributions [58,61,62]. Moreover, consistent with our affective tagging account, past work finds that 4-year-olds also use trait labels to make inferences about emotional reactions (e.g. expecting ‘nice’ children to be upset if their behaviour caused harm; [57]) suggesting that they may be using affective predictions over simple valence matching. Importantly, the extent to which children use behavioural distinctiveness and behavioural consistency across individuals and contexts shows considerable development between the ages of 3 and 6 [63]. It is typically not until 7 years of age that children expect consistency in behaviour over time [58,59,64]. Finally, the greater use of trait information (albeit on the unexpected non-social trait) in Study 1b relative to Study 2b is consistent with past findings demonstrating that children are biased towards positive information when evaluating others [65]. Specifically, if children are employing an affective tagging mechanism to select between social partners and are especially biased towards attending to positive information about others, it is less surprising that they struggled to identify a preferred partner in Study 2b. Our design followed from past work where children receive limited exposure to novel individuals before deciding where to invest their prosocial energies, which leaves open the possibility that children show different patterns of selectivity when interacting with individuals with whom they have richer histories (i.e. family, friends and peers; e.g. [66]). The results of these studies suggest an important avenue for future research involves examining under what circumstances, and when in development, children begin to display mature person preferences in the prosocial domain.

### 7.3. Limitations and future directions

Although this research provides an important extension to the extant literature, it is not without limitations. First, presenting the characters in vignettes allowed for methodological control and multiple trials. However, this ‘thin slice’ exposure is devoid of context and may have underestimated both adults' and children's person preferences. The theoretical frame for the current work is Trivers' theory of reciprocity [8] which is intended to explain the existence of cooperation between unrelated individuals. To that end, asking about person preferences in the context of limited information is the strongest test of the theory. Humans regularly make judgements about others’ characteristics, including trustworthiness and competence based on limited behavioural observation, or even the testimony of others (e.g. [67,68]). Moreover, once a trait has been attributed, humans tend to associate it with the individual across contexts and over time (e.g. [69,70]). That said, most human interactions do not exist in a ‘thin slice’ vacuum and typically involve rich histories of interaction and potentially conflicting cues (e.g. [44,66]). Relatedly, although both affective tagging and dispositional attributions involve *predictions* about how individuals will act in the future, we did not ask participants to make behavioural predictions. Instead, to avoid participants perseverating on the provided information, we asked them to choose with whom they would prefer to interact. Although previous work suggests that once children are able to make behaviour to trait attributions, they use this information to make accurate predictions (e.g. [57,63]) the way in which children are using affect (e.g. valence matching versus affective prediction) is an open question for future research.

A second concern relates to the comparability of contexts across the two samples. Because children tend to over-attribute goodness to lucky individuals [49,62] and because we wanted to ensure that children were not simply perseverating on their first selection over the three test questions, the general context in Studies 1b and 2b asked the children to assign preferred and unpreferred food to the two characters in each vignette. Although this assignment was non-costly, it is possible that the general context used with the children was more akin to common prosocial tasks (i.e. resource distributions) than the adult general context. Relatedly, it is possible that the two social tasks were not fully comparable in that choosing someone to play with at the park (Studies 1b and 2b) is likely more social than choosing someone to sit beside on the bus (Studies 1a and 2a). These concerns limit the strength of the conclusions we can draw from the overall lack of a trait by context interaction in the two developmental studies. Although it is important for future work to systematically probe the effect of context on children's social preferences, that children did not demonstrate a consistent preference for the prosocial trait, especially in the context of test questions that may have been construed as prosocial to varying degrees, is strong evidence that children are not using dispositional evaluations to select their prosocial partners.

Finally, all our participants were drawn from WEIRD samples [71], who are likely familiar with both empirical research and interacting with strangers. It is plausible that these patterns of preferences differ and develop along distinct trajectories in diverse cultural contexts where family units, peer groups and mobility vary [72,73]. Previous research has demonstrated that these are exactly the types of social behaviours that are influenced by socializing experiences (e.g. [74–76]). These limitations highlight directions for future research, particularly in understanding the associated social cognitive developments that lead to mature person preferences in the domain of prosocial behaviour, and the extent to which these preferences are influenced by culture.

### 7.4. Conclusion

The present research examined the development of the proximate evaluative mechanisms underlying prosocial partner choice. We found that adults appear to use dispositional attributions to identify individuals who are likely to repay prosocial investment. By contrast, children appear both less effective, and more general in their person preferences, using something more like affective tagging. Together these studies suggest that the mechanisms underlying prosocial partner choice are not early emerging and stable but instead show considerable development over childhood. Finally, these results highlight the need for additional research directly examining how individuals identify good social partners and the types of experiences that are required to develop these essential human skills.

## Data Availability

All materials, data and code are available in OSF: https://osf.io/e4zyf/. The data are provided in electronic supplementary material [77].
